# Sedentary Behavior Reduction: A Stepwise Approach to Increasing Physical Activity and Reducing Cardiovascular Disease Risk in Endometrial Cancer Survivors

**DOI:** 10.31083/j.rcm2307250

**Published:** 2022-07-07

**Authors:** Lauren C. Bates, Margaret I. Damare, Erik D. Hanson, Justin B. Moore, Victoria Bae-Jump, Michelle L. Meyer, Lee Stoner

**Affiliations:** ^1^Department of Exercise and Sport Science, University of North Carolina at Chapel Hill, Chapel Hill, NC 27599, USA; ^2^Human Movement Science Curriculum, University of North Carolina at Chapel Hill, Chapel Hill, NC 27599, USA; ^3^Lineberger Comprehensive Cancer Center, Chapel Hill, NC 27599, USA; ^4^Department of Implementation Science, Division of Public Health Sciences, Wake Forest University School of Medicine, Winston-Salem, NC 27101, USA; ^5^Division of Gynecology Oncology, University of North Carolina at Chapel Hill, Chapel Hill, NC 27599 USA; ^6^Department of Emergency Medicine, University of North Carolina at Chapel Hill, Chapel Hill, NC 27599, USA; ^7^Department of Epidemiology, Gillings School of Public Health, University of North Carolina at Chapel Hill, NC 27599, USA

**Keywords:** sedentary behavior, endometrial cancer, lifestyle behavior, cardiovascular disease, behavior change

## Abstract

Endometrial cancer survivors experience high rates of cardiovascular disease (*e.g., heart disease, obesity, diabetes*). The heightened cardiovascular disease risk may be attributed to cancer treatment coupled with sub-optimal lifestyle behaviors following treatment, including high amounts of sedentary behavior (SB). Public health agencies have graded the association of evidence between SB and cardiovascular disease as strong. However, while clinicians may wish to prescribe SB substitution strategies to reduce SB, guidelines do not currently exist. An additional challenge to behavior change pertains to the unique barriers that endometrial cancer survivors face, including treatment-associated fatigue and limited self-efficacy. Engaging in healthy movement behaviors, including minimizing SB and achieving recommended amounts of physical activity, are critical for health and well-being as well as cardiometabolic disease prevention. The purpose of this perspective paper is to propose an informed approach to physical activity promotion aimed to initiate movement and promote long-term behavior change by starting with an emphasis on reducing SB in endometrial cancer survivors. First, we address why endometrial cancer survivors should be targeted with SB reduction. Then, we suggest a stepwise approach to increasing physical activity by starting with SB reduction, including consideration to behavioral theories. Finally, we provide suggestions for future directions.

## 1. Introduction 

Endometrial cancer has one of the highest survival rates with 96% living at least 5 years post-diagnosis [1]. However, compared to the general population, endometrial cancer survivors experience a 3 to 6 fold greater risk of cardiovascular disease (CVD) compared to women without cancer [2, 3]. Moreover, CVD is the leading cause of death among early stage endometrial cancer survivors [4]. The heightened CVD risk can be attributed to obesity, metabolic syndrome [5], and sub-optimal lifestyle behaviors [3] such as high amounts of sedentary behavior (SB, defined as low-intensity activity in a seated/reclined posture with energy expenditure at or below 1.5 metabolic equivalents [6], Table 1, Ref. [6, 7, 8, 9, 10, 11, 12, 13, 14, 15]) and physical inactivity (defined as insufficient amounts of physical activity that is not meeting guidelines [16], Table 1). Compared to several other types of cancer survivors (breast, prostate, colorectal, and ovarian), prevalence of obesity and physical inactivity among endometrial cancer was the highest [17]. Engagement in healthy movement behaviors [7], including meeting recommended guidelines for physical activity [18] and minimizing sedentary behavior [19] are critical for health and well-being and the prevention of secondary disease [20, 21]. Physical inactivity and SB have been used interchangeably, however the two movement behaviors are distinct CVD risk factors [22, 23, 24, 25]. Interrupting SB with light physical activity has health benefits independent from moderate-to-vigorous physical activity [26, 27]. Yet, despite endometrial survivors spending the majority of their day in SB [28], survivorship programs have primarily focused on other modifiable health behaviors such as diet or physical activity [29, 30, 31, 32], and much less is known regarding the optimal prescription of SB [33].

**Table 1.** **Key terminology including definitions of endometrial cancer and cardiometabolic disease as well as movement behaviors and health behavior strategies**.

Key term	Definition	Reference
Endometrial cancer (EC)	Cancer of the endometrium (lining of the uterus). Cases are most commonly reported in women over 55 years of age. Symptoms such as abnormal vaginal bleeding often lead to early detection. If detected in early stages EC is highly treatable via surgery, chemotherapy, and/or radiation treatment.	[8]
Cardiometabolic disease (CMD)	Cardiometabolic diseases include cardiovascular disease, diabetes mellitus, and chronic renal failure. The cardiometabolic diseases represent a cluster of interrelated risk factors including hypertension, elevated fasting blood sugar, and abdominal obesity. Lifestyle risk factors also play a role in increasing CMD risk and include physical inactivity, smoking, and diet.	[9]
Lifestyle behaviors	Daily activities that are the result of an individual’s values, knowledge, and norms shaped by broad cultural and socioeconomic environment. Engagement in physical activity and sedentary behavior as well as nutritional behaviors (i.e., vegetable consumption) are examples of lifestyle behaviors. Healthy lifestyle behaviors decrease an individual’s risk for many chronic conditions including cardiovascular disease.	[10]
Movement behavior	Includes all physical activity, sedentary behavior, and sleep that occurs in a 24-hour cycle. Interaction between the three components of movement behavior patterns are associated with overall health and risk of chronic diseases such as cardiovascular disease.	[7]
Sedentary behavior (SB)	Any waking behavior characterized by an energy expenditure ≤1.5 metabolic equivalents (METs) while sitting, lying, or reclining. Examples of sedentary behavior: working at a desk, driving, watching TV, etc.	[6]
Physical activity (PA)	Any bodily movement produced by the contraction of skeletal muscle that increases energy expenditure above the resting metabolic rate (basal level). Types of physical activity include: exercise, occupational, conditioning, housework, etc. Physical activity is characterized by the **f**requency, **i**ntensity, **t**ime/duration, and **t**ype/modality. The characterization of PA is also known as the FITT principle.	[11]
Light-intensity physical activity	Any non-sedentary waking behavior that requires less than 3 METs. Examples: walking at a leisurely pace, light housework, and cooking.	[11]
Moderate-intensity physical activity	Activity that requires 3.0–5.9 METs. Examples: raking leaves and taking a brisk walk.	[11]
Vigorous-intensity physical activity	Activity that requires more than 6.0 METs. Examples: running, carrying heavy groceries upstairs, and strenuous fitness classes.	[11]
Physical inactivity	The term used to describe people who do not meet the recommended level of regular physical activity. Adults are considered physically inactive if they do not acquire at least 150 minutes of moderate-to-vigorous physical activity (MVPA) per week.	[11]
Theory of planned behavior	The theory of planned behavior suggests that an individual’s intentions to perform a certain behavior can be predicted and influenced by three main factors: (1) attitude toward the behavior, (2) subjective norms, and (3) perceived behavioral control.	[12]
Implementation intentions	Synonymous with “if-then” planning, implementation intentions help bridge the gap between behavioral intention and actualization. Formulation of implementation intentions involves planning out when, where, and how goal-directed behaviors will be enacted to help individuals avoid self-regulatory pitfalls on the path toward goal realization. Setting implementation intentions promotes initiation and continuation of goal-directed behaviors even when faced with unwanted influences. An example of setting implementation intentions, “When my phone beeps three times, I will stand up for 30 seconds.”	[13]
Mental Contrasting	A self-regulatory strategy that helps build strong goal commitment via increasing perceived self-efficacy. Mental contrasting involves comparatively visualizing a desired outcome followed by the realistic obstacles.	[14]
Intention-Behavior Gap	The failure to translate a goal intention into an actualized behavior.	[15]

A working hypothesis is that SB contributes to CVD risk via repeated exposure to acute sitting-induced increases in arterial stiffness likely driven by hemodynamic changes with metabolic, autonomic, and hormonal factors [25]. During acute prolonged sitting, a lack of muscle pump activity leads to lower extremity venous blood pooling and reduced blood transit time in venous circulation, which subsequently decreases venous return inversely with stroke volume [25, 34]. Reduced stroke volume decreases shear stress which promotes oxidative stress, endothelial dysfunction, and acutely increases arterial stiffness [35, 36, 37, 38, 39]. Overtime with repeated exposure this may result in structural remodeling of vessel walls [25, 40] potentially explaining the positive association between chronic SB and arterial stiffness [41] and between SB and CVD [42, 43]. The purpose of this paper is to propose an informed approach to physical activity promotion aimed to initiate movement and sustain long-term behavior change by starting with an emphasis on reducing SB in endometrial cancer survivors. Specifically, we will (i) present key terminology (Table 1) and discuss existing literature examining SB in endometrial cancer survivors, (ii) present a stepwise approach to reducing SB, (iii) propose considerations for research on the stepwise approach, and (iv) conclude with practical implications.

## 2. Risk for CVD and Potential Role of Activity Behaviors 

### 2.1 Endometrial Cancer Survivors are at High Risk for CVD

Endometrial cancer is the 4th most commonly diagnosed cancer in women [44], and with 5-year survival exceeding 90% [1] there is a large population of survivors. However, endometrial cancer survivors are more likely to die from CVD then their cancer [4]. Furthermore, unlike many other cancers, endometrial cancer incidence is growing with more cases being diagnosed every year likely due to the increasing obesity epidemic, with many shared risk factors between endometrial cancer and obesity [45, 46]. Factors that contribute to obesity risk (*i.e., insulin resistance*) are also associate with endometrial cancer risk [47]. However, risk of death from CVD is greater than risk of death from endometrial cancer five years after diagnosis with an age-adjusted standardized mortality ratio of 8.8 (95% confidence interval 8.7–9.0) [3, 48]. In a Surveillance, Epidemiology, and End Result (SEER) study, Ward *et al*. [3], report that over time the probability of CVD increases in endometrial cancer survivors and calls for investigation into potential survivorship interventions aimed at targeting CVD risk reduction.

### 2.2 Endometrial Cancer Survivors and Poor Activity Behaviors

An estimated 80% of survivors of endometrial cancer are overweight/obese and spend a large amount (>8 hours per day) of time sedentary [28, 49], posing a significant threat to their well-being and health-related quality of life [50]. However, existing literature investigating SB in endometrial cancer survivors is currently limiting by data only including questionnaire (not objective accelerometry data) [51]. The majority of survivors exhibit the motivation to change these patterns of behavior in wake of their cancer diagnosis [50] but only about half of cancer survivors in general receive consultation on improving activity behaviors [52]. Furthermore, it is not clear why endometrial cancer survivors are sedentary. There is a moderate level of evidence linking sitting time and endometrial cancer [53], but significantly less evidence pertaining to why endometrial cancer survivors are sedentary and the behavioral determinants influencing activity behaviors. Information regarding SB in endometrial cancer survivors is currently limited by the majority of the evidence being cross-sectional survey data. More research is needed to better understand this multidimensional behavior in endometrial cancer survivors as each cancer survivorship group has unique needs depending on the biology of the disease, patient characteristics, and effects of treatment [54]. For example, early-stage endometrial cancer is curative with surgery yet patients (who are likely obese and have multiple comorbidities) [54] need survivorship interventions improve lifestyle behaviors to improve cardiometabolic disease burden.

Despite substantial evidence of the benefits of physical activity such as CVD risk reduction and disease prevention [55], participation is extremely low. An estimated 80% of US adults do not meet recommended physical activity levels (≥150 mins of moderate-to-vigorous intensity physical activity per week) [56] likely due to barriers such as lack of time, access, or knowledge [57, 58]. One study of N = 120 early-stage endometrial cancer survivors found that 88.3% were physically inactive [54] despite physical activity being correlated with quality of life in endometrial cancer survivors [59]. We hypothesize that the existing physical activity interventions do not account for the specific challenges that survivors face such as low physical function, obesity, fatigue, and neuropathy (Table 2) [60, 61, 62, 63, 64]. Participation in physical activity is even lower in clinical populations such as those with endometrial cancer who are burdened with barriers and challenges from cancer treatment such as low physical function, pain, obesity, neuropathy, and fatigue [60, 61, 62, 63, 64]. As physical inactivity and SB are distinct risk factors [65], individuals meeting recommended guidelines for physical activity engagement (150 min/week) are not fully protected from the detrimental cardiovascular effects associated with SB.

**Table 2.** **Challenges endometrial cancer survivors face to healthy lifestyle engagement and how sedentary behavior interruption may address these concerns**.

Barriers to physical activity engagement	Consequences of barriers for endometrial cancer survivor	How sedentary behavior interruption strategies address barriers
Obesity	Restricts movement, limits fitness, joint stress	Low skill, limited exertion, closed chain movements to limit joint strain and reduce risk of injury
Low physical fitness	Chronic disease risk, limited muscular strength and endurance, low cardiorespiratory fitness	Utilizes low MET activities of daily living—walking or standing—to avoid overexertion, incrementally increase fitness, and improve quality of life
Fatigue	Reports of being too tired to exercise, mental health impact (feeling discouraged), contributes to sedentary behavior	Requires less time than exercise, uses movements that are already needed in daily life, and limits exertion
Pain	Restricts movement, limits fitness, contributes to sedentary behavior	Utilizes low impact, modifiable movements to minimize stress on the body
Neuropathy	Restricts movement, contributes to balance challenges	Can be completed with physical support (i.e., walker or counter-top for balance), relieves pressure in the lower extremities. Can involve predominantly static activities (i.e., standing) = reduces the need for physical support in patients with neuropathy in their upper extremities
Low self-efficacy	Lack of motivation and lack of self-belief	Utilizes movements requiring a low skill level and minimal instruction which increases confidence in one’s ability to complete task
Limited physical activity experience	Lifetime of physical inactivity, previous poor experience with physical activity, chronic poor lifestyle movement behaviors	Easier to start standing and/or walking than engaging in exercise, can be completed at home with little resources

There is a growing presence of both movement behaviors (physical activity and SB) in public health guidelines [66]. However, the recommendations for reducing SB are far less specific vaguely stating “sit less and move more” [66]. There are critical gaps in SB interruption recommendations that need to be filled including frequency, intensity, time (duration), and type (modality such as standing or walking). Our best evidence for breaking up SB to reduce CVD risk includes interruptions every 20–30 minutes using a light intensity activity like walking for 2–5 minutes [67]. However, existing evidence does not account for special populations, like endometrial cancer survivors, who may need tailored recommendations and have greater risk for CVD [4]. For example, the ACSM physical activity guidelines provide specific recommendations for special populations like cancer survivors, who made need different SB interruption prescription (frequency, dose, type) but more SB research is needed before we have enough evidence to create such prescription. Furthermore, current guidelines do not provide recommendations addressing promotion from SB interruption to regular engagement in physical activity. The progression from reducing to SB to meeting physical activity guidelines is critical for individuals to reap the benefits of physical activity. However, current guidelines do not include progression strategies.

### 2.3 Activity Behavior Interventions in Endometrial Cancer Survivors Have Had Limited Success

Despite strong evidence of endometrial cancer survivors engaging in high amounts of SB [50], there is a clear lack of interventions targeting SB reduction. Additionally, there are limited physical activity interventions targeting endometrial cancer survivors. Existing literature reports improvement in quality of life following physical activity (*i.e., walking*) interventions [63, 68, 69, 70], but concerningly there are frequent reports of adherence and compliance [68, 70] issues even greater than the general population. Furthermore, a qualitative study conducted by Koutoukidis *et al*. [71] reported endometrial cancer survivors experience many challenges and barriers to physical activity engagement (i.e., time, financial, and geographical constraints; treatment effects; obesity-related stigma social; and a lack of information/instruction) and express a clear lack of knowledge on what activity they should be trying to complete (frequency, intensity, time, and type).

In terms of SB reduction interventions, findings in individuals with obesity can guide our understanding regarding some of the unique challenges endometrial cancer survivors face. For example, Judice *et al*. [72] conducted a pilot study to evaluate the short-term effectiveness and feasibility of prompting SB interruption in individuals with obesity throughout the workday and during home/leisure time activities. Results indicated the intervention strategy was successful in reducing SB and increasing the amount of time spent standing/walking per day. Upon review of participant feedback, it was clear that participants responded to the prompts by increasing the length of standing/light physical activity bouts and preferred this time adjustment to more frequent interruptions in SB [72]. The authors reported behavioral resistance to more frequent sit to stand transitions. It appears participants with obesity do not prefer more frequent SB interruptions (with a shorter duration), but instead would rather increase the duration of the interruption with walking or standing (decreasing frequency of interruption). In a population of older adults, Hartman and colleagues [73], conducted a long-term (16 weeks) SB intervention study during which, the intervention was adjusted based on participant feedback to intensify coaching and support during the latter portion of the study. These adjustments ultimately led to a significant decrease in SB and increase in PA (light intensity) among individuals at high risk for CVD. A strong foundational understanding of the needs of the population is critical to support feasibility, since an intervention will only work if participants are willing to adhere to it. Design components aimed to promote long-term adherence to SB interventions in special populations (e.g., endometrial cancer survivors) are lacking and constitute a methodological pitfall common in SB intervention design and study approach. The results from Judice *et al*. [72] and Hartman *et al*. [73] emphasize that active inclusion of participant feedback is important for the development and modification of feasible interventions suited to the target population that will facilitate easy habituation and long-term adoption of the intervention [72].

### 2.4 Endometrial Cancer Survivors Face Unique Challenges to Activity Engagement 

Endometrial cancer survivors face many challenges to engaging in healthy movement behaviors such as low physical function [63], cancer related fatigue [60, 61], obesity [62], pain [63], and limited self-efficacy [64]. Compared to other types of cancer survivors, endometrial cancer survivors in particular engage in higher amounts of SB [53]. Endometrial cancer survivors are also highly inactive [74]. Evidence suggests that physical activity engagement provides clear benefits for CVD prevention and survivorship such as enhanced quality of life [68]. However, only an estimated 12–29% of endometrial cancer survivors meet physical activity guidelines [59, 75]. Reducing SB may be a feasible strategy to reduce CVD burden in endometrial cancer survivors because interruptions strategies such as standing or walking may be more feasible for this population that is commonly both deconditioned and overweight/obese [76]. Additionally, SB reduction likely requires fewer resources (e.g., education, cost, time) than changing other CVD related lifestyle behaviors (e.g., physical activity, diet, sleep). When aiming to improve lifestyle behavior in endometrial cancer survivors, reducing SB may be a critical first step that may lead to subsequent physical activity engagement.

## 3. A Stepwise Approach to Increasing Physical Activity 

### 3.1 A Proposed Stepwise Approach to Improving Activity Behaviors in Endometrial Cancer Survivors

**Fig. 1.**
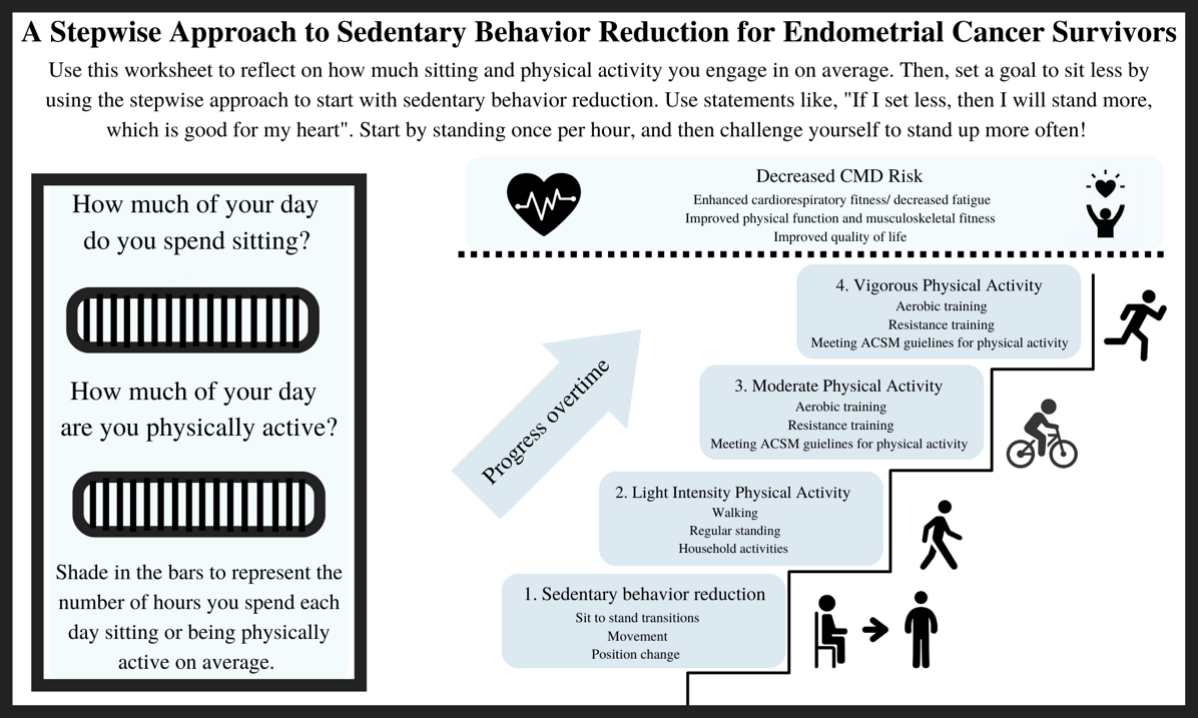
**Stepwise Approach to Sedentary Behavior Reduction for Endometrial Cancer Survivors**. This figure represents a worksheet that could be distributed to endometrial cancer survivors when they are waiting for their appointment to start in clinical settings. The endometrial cancer survivor will self-reflect, and shade in the circles to represent how much time they spend sitting or being physically active per day. It also gives examples of goal setting, and provides easy to follow steps to increase physcial activity by starting with sedentary behavior reduction.

As the prevalence of CVD in endometrial cancer survivors continues to increase, it is necessary to move beyond “sit less and move more” in an effort to prescribe specific guidance to reduce SB [56]. Recent research [66, 77] into preventative measures has increasingly focused on the relationship between SB and chronic disease. This includes exploring the impact of SB reduction on disease risk independent of and in conjunction with physical activity interventions. Dogra *et al*. [78], suggests an approach to clinical physical activity counseling that starts with reducing SB. The proposed stepwise approach recommends clinicians begin with motivational interviewing designed to assess their patients’ current SB and physical activity habits along with their motivation and ability to change said habits (Fig. 1). If clinician time constraints limit the feasibility of implementation, then perhaps they could provide a referral (i.e., to see a nutritionist and/or physical therapist) or handout such as Fig. 1 instead. However, provider recommendations regarding diet and exercise are associated with beneficial changes in cancer patients’ lifestyle behaviors [79]. For patients who are sedentary and/or do not meet physical activity guidelines, the stepwise approach [78] suggests healthcare teams (*i.e., clinicians, researchers, nurses, physician assistants, physical therapists, psychologists, etc*.) begin with SB counseling. Since endometrial cancer survivors face additional challenges to healthy lifestyle engagement, and individuals with obesity display behavioral resistance to frequent SB interruptions [72], we suggest the stepwise approach to increase physical activity should begin by targeting SB once per hour.

The goal of stepwise SB counseling is to interrupt prolonged SB and increase the amount of sit to stand transitions (ideally, 2–5 per hour) [78]. Since changing lifestyle behaviors may be met with behavioral resistance, theoretically informed behavioral counseling approaches employing goal-setting [78] and mental contrasting [80] need to be prioritized. Increasing the duration of the interruption appears to be the first step then individuals should work to increase the frequency of the SB interruptions over time [72]. Once the individual has successfully adopted the goals of step one (increased sit to stand transitions) into their daily routine, the clinician/researcher/healthcare team member will help them move on to and through step two which involves participation in light intensity physical activity (*i.e., walking, household chores, gardening, playing with pets*). The third step involves a progression towards incorporating moderate physical activity (*i.e., resistance training, brisk walk, jog, biking*) and the fourth step involves vigorous physical activity (*i.e., running, jumping, swimming, heavier resistance training*). The final step includes reaching a level of cardiorespiratory and musculoskeletal fitness that is associated with positive overall health outcomes such as reduced CVD risk. In the final step, maintenance of recommended physical activity and SB reduction will be achieved. By beginning with reducing SB, the stepwise approach [78] addresses many of the most commonly cited barriers to physical activity among endometrial cancer survivors, including physical limitations to exercise, fatigue, time constraints (interrupting SB requires less time than traditional physical activity/exercise engagement), transportation (SB reduction can occur at home or at work) [56]. The stepwise approach [78] to SB reduction is also grounded in behavioral theory, potentially making it a feasible strategy to use in endometrial cancer survivors [80].

### 3.2 Considerations for Research on Stepwise Approach

The overarching goal of research should be to inform policy that provides clear guidelines to promote health and well-being. Current guidelines for SB reduction are lacking in specificity and fail to include special populations that are at higher risk for CVD such as endometrial cancer survivors. Endometrial cancer survivors are amenable to lifestyle changes during follow-up care [17, 81]. However, these patients struggle to successfully implement lifestyle changes to improve their health-related quality of life and increase long-term survivorship. A recent study conducted by the American Society of Clinical Oncology (ASCO) found that only 56.87% of respondents reported discussion about exercise during visits to their oncologists, thus it is likely that many cancer patients are unaware of their inactivity and its impact [79]. Thus, in adapting SB and physical activity interventions for endometrial cancer survivors, it is crucial to eliminate as many of the population-specific challenges to facilitate long-term behavior change. For example, handouts and conversations with their oncologist about diet and exercise serve to educate cancer patients about PA and SB as well as direct them toward helpful resources. When researchers and clinicians design interventions utilizing the stepwise approach, behavioral theories to promote enhancing self-efficacy will likely make long-term maintenance of healthy lifestyle behaviors more feasible. A recent meta-analysis reports a significant relationship between an individual’s confidence in their ability to reduce SB and lower levels of SB (combination of objective and self-reported SB measurement) [82] suggesting that self-efficacy may be a critical factor in reducing SB in endometrial cancer survivors. Increases in self-efficacy has resulted in initiating leisure-time walking within sedentary women after and implementation intentions intervention [83].

Implementation intentions aim to promote initiation of goal-oriented behaviors, encourage behavioral maintenance when presented with unwanted obstacles, and promote accessibility and automaticity of goal-directed responses [84]. Implementation intentions (or making “if-then” plans) should be used in conjunction with mental contrasting, a self-regulatory strategy, the intended goal with realistic obstacles [84]. “If-then” plans foster sustained behavioral change by creating goal-directed behaviors and attaching them to external cues which ultimately promotes initiation and automaticity of these behaviors (*i.e., “When my phone beeps three times I will stand up for 30 seconds.”*). Mental contrasting is also important in creating effective implementation intentions [14] because it preemptively creates an achievement-focused mental structure for approaching adversity and promotes a goal-oriented response to unwanted influences/obstacles. These behavioral techniques should be integrated into the first step of the stepwise approach (Fig. 1) and then maintained throughout to increase physical activity by starting with SB reduction.

The lack of success in translating SB interventions from non-clinical populations to endometrial cancer survivors can be described as a result of the intention-behavior gap [85]. The gap between goal intentions and goal achievement is common problem inhibiting behavior change in all of us to some extent. The intention-behavior gap is often a result of multiple interacting factors [85]. In the case of endometrial cancer survivors, the gap between strong goal intentions—to change physical activity and/or SB—and long-term lifestyle changes is due, in large part, to a disproportionate number of obstacles that are not accounted for in typical physical activity interventions. Endometrial cancer survivors need a behavioral approach to increase self-efficacy and increase their resilience against obstacles in order to implement lifestyle behavior change. Successful integration of behavioral techniques into—properly adapted—SB interventions for endometrial cancer survivors will promote initial behavior change and progress maintenance. Therefore, we suggest a stepwise approach [78] to reduce SB will successfully lead to increased physical activity long-term compared to traditionally implementing physical activity without consideration for behavior chance to maintenance.

## 4. Conclusions

Endometrial cancer survivors experience high risk of CVD due to cancer treatment and sub-optimal lifestyle behavior such as high amounts of SB and physical inactivity. Challenges to healthy lifestyle engagement such as fatigue, obesity, low physical function, pain, and low self-efficacy have led to limited success in interventions improving lifestyle activities. To successfully motivate lifestyle behavior, change and to enhance self-efficacy in endometrial cancer survivors, behavioral strategies should be integrated in the development of interventions. This paper presents a stepwise approach that could be implemented by providers to increase sustainable physical activity overtime. Although future research is needed to test the effectiveness of a stepwise approach to SB reduction in endometrial cancer survivors, we hypothesize that a stepwise approach starting with SB reduction supported by behavioral theories may be a feasible strategy to lead to long-term physical activity engagement for endometrial cancer survivors.
